# Relative enlargement of the medial preoptic nucleus in the Etruscan shrew, the smallest torpid mammal

**DOI:** 10.1038/s41598-022-22320-y

**Published:** 2022-11-03

**Authors:** Senmiao Sun, Michael Brecht

**Affiliations:** 1 Neural Systems and Behavior, Marine Biological Laboratory, 7 MBL Street, Woods Hole, MA 02543 USA; 2 Department of Neurobiology, Harvard Medical School, Boston, MA 02115 USA; 3 Program in Neuroscience, Harvard Medical School, Boston, MA 02115 USA; 4 Bernstein Center for Computational Neuroscience Berlin, Humboldt-Universität Zu Berlin, Philippstr. 13, Haus 6, 10115 Berlin, Germany

**Keywords:** Neural circuits, Hypothalamus, Circadian regulation

## Abstract

Endothermy is a key feature of mammalian biology and enables mammals to maintain stable body temperature and homeostatic functions in the face of a rapidly changing environment. However, when faced with harsh environmental conditions, certain mammalian species enter torpor, a state characterized by reduced metabolism, body temperature, and activity, to minimize energy loss. Etruscan shrews are the smallest mammals, with a surface-to-volume ratio that is very unfavorable for endothermic animals. As a result, Etruscan shrews have an extremely high metabolic rate and are known to enter torpor frequently, presumably as an energy-saving measure. Despite the recent identification of medial preoptic area (MPA) as a key brain region to regulate torpor in mouse, little is known about neural control of torpor in other endothermic animals, including the Etruscan shrew. Here, we confirmed that Etruscan shrews readily enter torpor even in the absence of strong physiological triggers. We then compared the medial preoptic nucleus (MPN) within the MPA of Etruscan shrew and rat, a mammal that does not enter torpor under physiological conditions. While rats have roughly 100 times the body weight and 33 times the brain weight of Etruscan shrews, we find that the male rat MPN exhibits only 6.7 times the volume of that of the male Etruscan shrew. Accordingly, the relative brain volume of the MPN was 6.5-fold larger in shrews, a highly significant difference. Moreover, MPN neuron counts were only roughly twofold lower in shrews than in rats, an astonishing observation considering the interspecies size difference and that neocortical neurons are ~ 20 × more numerous in rats than in shrews. We suggest that the extraordinary enlargement of the Etruscan shrew MPN is a specialization for orchestrating torpor in a mammal with an exceptional metabolism.

## Introduction

The advent of endothermy in mammalian species has been considered as one of the most critical points in vertebrate evolution, as maintaining a high body temperature maximizes many physiological functions, including digestion, mobility, and brain function^1^. However, maintaining a constant body temperature that is higher than the ambient temperature costs a huge amount of energy and requires a comparable increase in food supply. When faced with cold ambient temperature and a shortage in the food supply, some heterothermic mammalian species and birds evolved alternate strategies^2^, such as daily torpor and hibernation, to avoid the energy costs of maintaining a high body temperature. Unlike hibernation, which is seasonal and usually spans weeks to months, daily torpor lasts for a few hours and is often interrupted by food foraging^3^. Upon entry into torpor, animals dramatically reduce their metabolic rate, resulting in a decrease in core body temperature and activity at the same time. Recent studies in mice^4–6^ identified the medial preoptic area (MPA) as a brain region critically involved in controlling fasting-induced torpor. However, whether this area is crucial for regulating torpor in other mammalian species and how it might differ in non-torpid species remains unknown.

Etruscan shrews are the smallest mammal by mass, with a large surface-to-volume ratio that makes them easily subject to heat loss. Therefore, Etruscan shrews have an exceptionally high metabolic rate of up to 1511 heart beats per minute^7,8^. To maintain such high metabolism, Etruscan shrews need to consume food about 1.5–2 times their body weight per day. However, when the food supply becomes inadequate, Etruscan shrews are known to enter torpor frequently, presumably as an energy-saving measure^9^.

To begin to address whether the MPA could serve to regulate torpor in Etruscan shrews, we compared the medial preoptic nucleus (MPN) within the MPA of Etruscan shrew and rat, a mammal that does not naturally enter torpor. In Nissl-stained sections, the MPA could be unambiguously identified in both species as a cell dense region below the crossing of the anterior commissure, but the MPN was more anatomically distinct in Etruscan shrews than in rats due to extraordinarily dense cell body packing in this region. Indeed, we observed that the relative brain volume of the MPN is significantly larger in Etruscan shrew than in the rat. Despite 20 times more neocortical neurons in rats than in Etruscan shrews, we found that Etruscan shrew MPA neuron counts are only twofold less than those of rats, a remarkable difference. We conclude that the enlarged MPA in Etruscan shrews might serve as the key brain region to regulate torpor and speculate that this relative enlargement may contribute to explain why Etruscan shrews could enter torpor, while rats do not.

## Results

**Figure 1:**
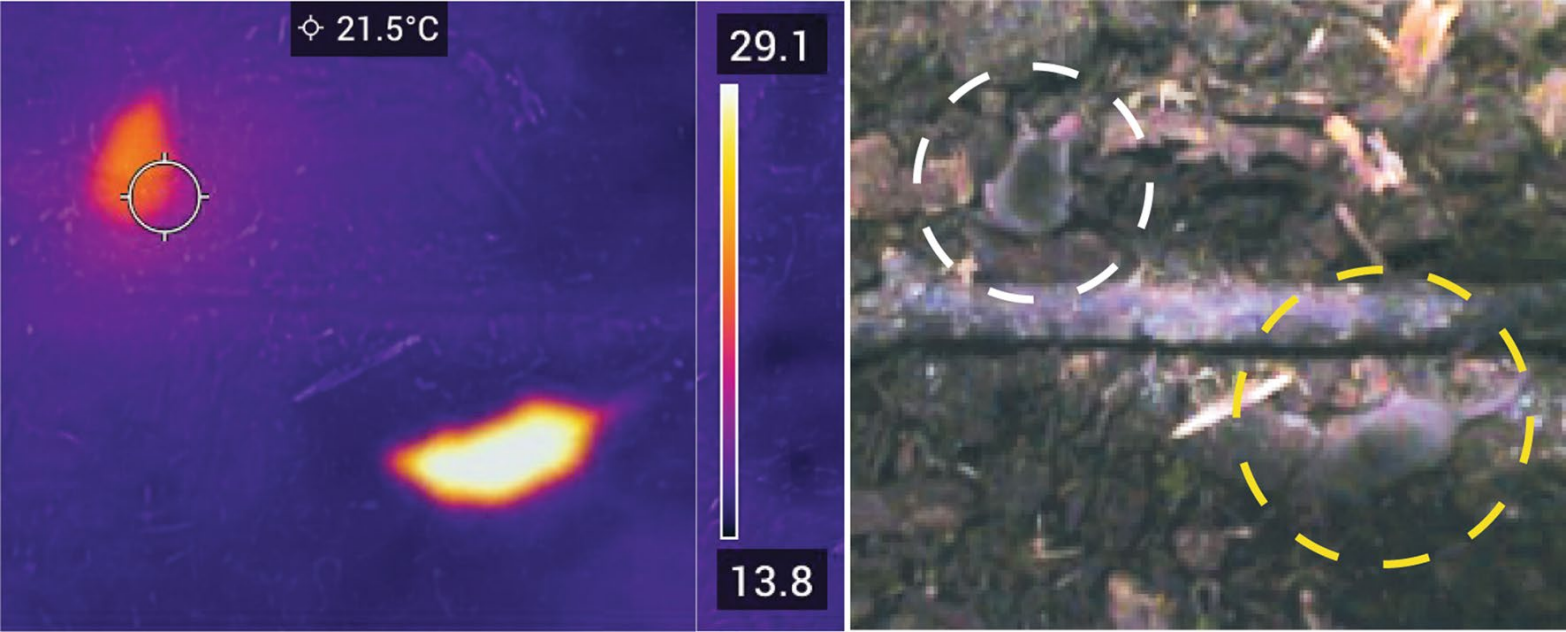
Etruscan shrews readily enter torpor upon fasting. Thermal image and color photograph of a normally active shrew (yellow dashed circle) and a torpid shrew (white dashed circle). The shrew in torpor shows a surface body temperature of approximately 22 °C, as compared to ~ 29 °C for the non-torpid animal.

Etruscan shrews have a highly specialized metabolism and readily enter torpor^9^. To confirm this tendency of Etruscan shrews to enter torpor, we housed male shrews in a reverse 12 h light/dark cycle chamber at room temperature and fasted them at the beginning of the dark cycle (their normal feeding intervals). We found them often to be unresponsive at the end of the daily feeding intervals. Thermal imaging suggested a dramatic drop in body temperature in such unresponsive animals, a hallmark of torpor (Fig. 1). After disturbing such unresponsive animals, we observed them to shiver before resuming their normal activity.

**Figure 2:**
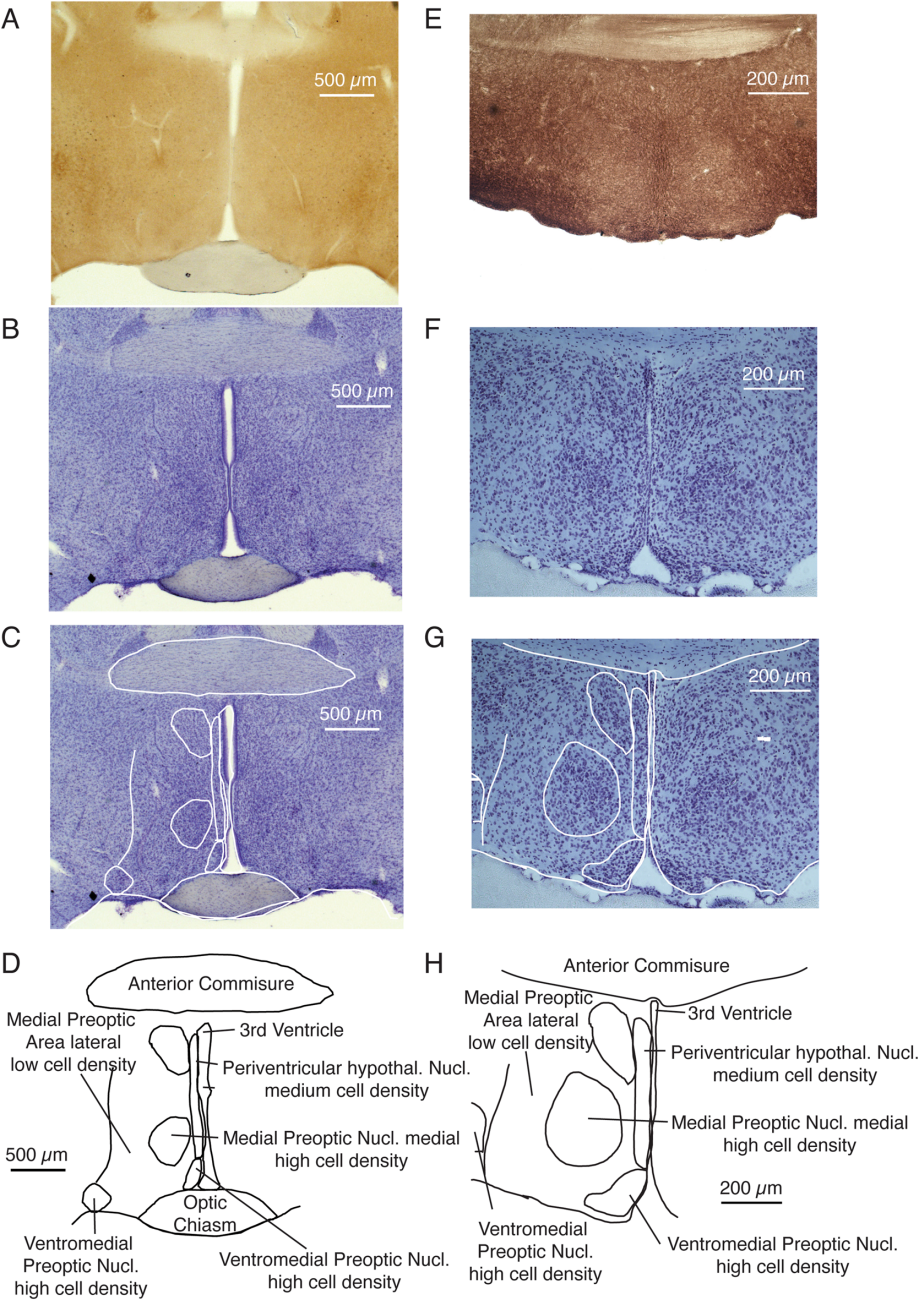
Identification of medial preoptic nuclei in rats and Etruscan shrews. (**A**) Rat coronal brain section of the medial preoptic area stained for cytochrome oxidase activity. The staining pattern is relatively indistinct. (**B**) Rat coronal brain section containing the MPA of rat stained for Nissl substance. Variations in cell density indicate different medial preoptic nuclei. (**C**) Same section as above with cytoarchitectonic divisions highlighted by white lines. (**D**) Cytoarchitectonic divisions named according to the Paxinos and Watson nomenclature. Our further analysis focused on the Medial preoptic nucleus (MPN). (**E**) Etruscan shrew coronal brain section of the medial preoptic area stained of cytochrome oxidase reactivity. The medial part of Medial preoptic nucleus stands out by a lower cytochrome oxidase reactivity (whitish). (**F**) Etruscan shrew coronal brain section containing the MPA of rat stained for Nissl substance. Variations in cell density indicate different medial preoptic nuclei. (**G**) Same section as above with cytoarchitectonic divisions highlighted by white lines. (**H**) Cytoarchitectonic divisions named according to the Paxinos and Watson nomenclature. Note the distinct medial preoptic nucleus.

Given recent findings implicating the MPA in the control of rodent torpor, we sought to investigate whether the MPA of a torpor-prone species such as the Etruscan shrew harbors distinctive features compared to a species that does not naturally enter torpor such as the rat. To this end, we performed staining for cytochrome oxidase reactivity (Fig. 2A) and Nissl substance (Fig. 2B) in coronal sections of the rat brain. While cytochrome oxidase reactivity was relatively indistinct in the MPA of rat, cytoarchitectonic divisions were clear in the Nissl stain (Fig. 2C) and we detail the nomenclature and cytoarchitectonic features of these divisions in the MPA of rat in Fig. 2D. Our further analysis focused on the MPN. This nucleus was distinctly identified by a low cytochrome oxidase reactivity in the MPA of Etruscan shrew (Fig. 2E). In Etruscan shrews, much like in rats, cytoarchitectonic divisions were clear in the Nissl stain (Fig. 2F,G) and we detail the nomenclature and cytoarchitectonic features in Fig. 2H.

**Figure 3:**
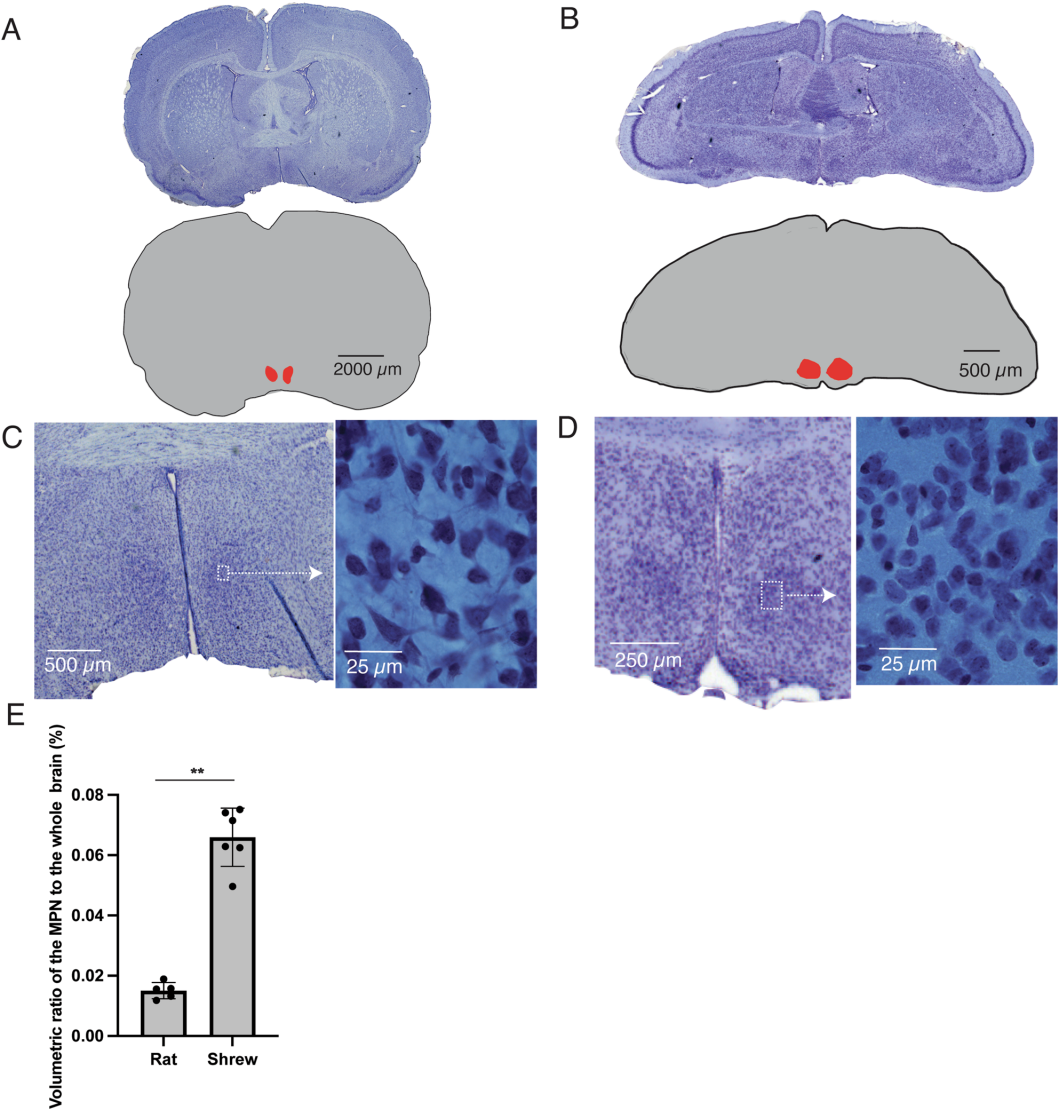
Etruscan shrews have a relatively enlarged MPN volume compared to rats. (**A**) Upper, rat coronal brain section containing the MPA of rat stained for Nissl substance. Lower, schematic drawing of the same section, with the MPN highlighted in red. (**B**) Brain section and MPA of the Etruscan shrew, conventions as in (**A**). (**C**) Left, higher magnification (10x) view of a rat brain section showing the MPN. Right 100 × magnification to show individual neurons within the MPN. (**D**) MPN of an Etruscan shrew, conventions as in (**C**). (**E**) Bar graph showing the percent volume of the MPN to the volume of whole brain in male rat and male Etruscan shrew, **p < 0.01, Mann–Whitney *U* test. Volumetric measures involved five male rats and six male shrews.

In our rat Etruscan shrew comparison we focused on samples only from male shrews and rats because MPN is sexually dimorphic and has different sizes in male and female anmials^10^. To ensure the consistency of measurements across animals, we started measuring the MPN when the anterior commissure merges and the MPN becomes distinctive. We stopped measurements when the anterior commissure starts to vanish and the MPN becomes less anatomically defined and disappearing (Fig. 3A,B). Using these parameters the measured MPNs usually span ~ 200 µm anteriorly-posteriorly in the Etruscan shrews and ~ 500 µm in rats. We found that the rat MPN exhibits 6.7 times the volume (0.18 ± 0.03 mm^3^) of that of the shrew (0.027 ± 0.004 mm^3^). However, the relative brain volume of the MPN to the volume of whole brain was 6.5-fold larger in shrews, a highly significant difference (p = 0.004, z = -2.8, Mann–Whitney *U* test) (Fig. 3E). Moreover, in the shrew the MPN is extremely cell dense, making the region more clearly distinguishable from surrounding areas than in the rat (Fig. 3C,D).

**Figure 4:**
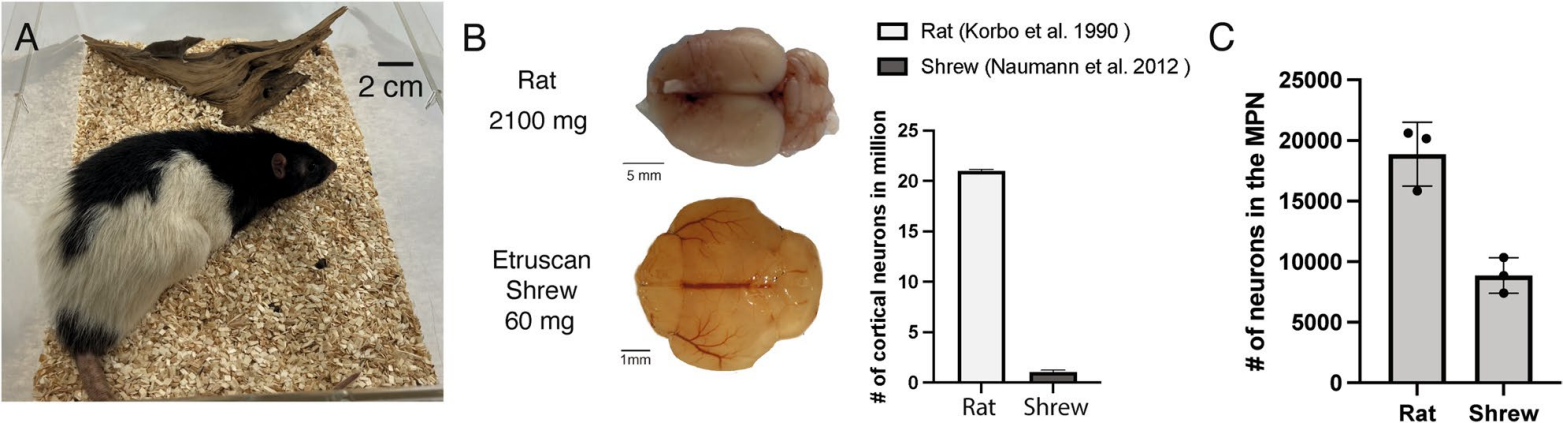
Neocortical and MPN neurons count in rats and Etruscan shrews. (**A**) Comparison of the body size between adult male Etruscan shrew and male rat (6-month-old). (**B**) Left, rat and Etruscan shrew brains. Right, the rat brain contains about 20 × more cortical neurons than that of the shrew. Exact numbers extracted from (Naumann et al. 2012; Korbo et al. 1990)^11,12^ and illustrated as a bar graph. (**C**) The rat brain contains only about 2 × more MPN neurons compared to the shrew. Cell counts were performed on three male shrews and three male rats and refer to the sum count of neurons in both hemispheres.

Etruscan shrews and rats are of remarkably different size (Fig. 4A). In this regard, the rat brain weight is roughly 33 times that of Etruscan shrew and contains 20 × more cortical neurons (Fig. 4B, data adapted and replotted based on published studies Korbo et al. 1990 & Naumann et al. 2012)^11,12^. However, MPA neuron counts were unexpectedly only roughly twofold lower in shrews (8860 ± 1467 SD) than in rats (18,871 ± 2630 SD) (Fig. 4C). We therefore conclude that this relative enlargement of the MPA in Etruscan shrew may serve to support its high metabolism and torpor tendency.

## Discussion

Torpor and hibernation have evolved to protect endothermic animals from harsh environmental conditions. Despite decades of research characterizing the physiological changes during torpor and hibernation, very little is known regarding the central mechanisms that regulate such behaviors. Identification of the preoptic area as a hub for regulating mouse torpor has provided an entry point to dissect the neuronal circuits that drive torpor and presumably hibernation as well. In this study, we investigated torpor behavior in Etruscan shrews. Unlike a previous study that reported that the induction of torpor in the Etruscan shrew requires both fasting and cold environment exposure^13^, we found that fasting alone is sufficient to drive prominent torpor in this species, with surface body temperature dropping close to ambient temperature. Given the ease of torpor induction, we therefore propose that Etruscan shrew could also serve as a model to study torpor-related behavior.

Notably, we found that the medial preoptic nucleus is relatively enlarged in the shrew compared to that of the rat, a non-torpid animal. The relative increase in MPA tissue volume and neuronal number in the shrew may serve to regulate the animal’s high metabolic needs and torpor behavior even though a direct correlation could not be established. Future studies could utilize loss-of-function approaches to further confirm the functional importance of the MPA in Etruscan shrew. Given the sexual dimorphism of MPA and torpor behavior, we only examined torpor behavior in male shrews. It would also be of interest to test if differences in relative MPN volumes and neuronal number hold in other torpid and non-torpid species, for example in the Gray Mouse Lemur, a small primate that also can enter torpor^14^. Moreover, since the cell types that regulate torpor in the MPA have been identified^4–6^, it would be beneficial to examine the molecular composition of the MPA in other torpid animals, like the Etruscan shrew, through modern sequencing and in situ hybridization approaches. Combined with additional molecular analyses, cross-species study of the MPA could be used to identify circuits that might be manipulated in naturally non-torpid species to induce a torpor-like state^6,15^. Eventually, such studies could help facilitate the induction of synthetic torpor^16^ in humans, which has significant potential medical applications as well as relevance for enabling extended space exploration.

## Materials and methods

### Animals and tissue

Etruscan shrews used in this study were housed in terraria containing a layer of dry soil, moss, and broken flowerpots. Crickets were provided as a food source, with water ad libitum. The detailed housing conditions were as previously described^17^. The adult rat (6 months) photographed next to the stuffed shrew was used in another experimental study and was obtained from Janvier Laboratories (Le Genest-Saint-Isle, France).

For coronal brain slices, animals were euthanized by isoflurane and then perfused transcardially with 0.9% saline followed by 4% paraformaldehyde in 0.1 M phosphate buffer (PB). After postfixation in 0.1 M PB, the brains were immersed in 30% sucrose solution for cryoprotection until they sank to the bottom of the vial. All brains were embedded in a mixture of egg yolk, and 30% sucrose supplemented by 0.75 mL glutaraldehyde and mounted on a cryostat (Lecia 2035 Biocut) to obtain 30- or 40-µm-thick coronal sections. Brains were then mounted on glass slides for Nissl staining^11^. Sections were stained for cytochrome-oxidase activity using the protocol of Wong-Riley (1979)^18^.

### Torpor behavior

Etruscan shrews were housed in a reverse 12 h light/dark cycle for convenience. Torpor occurred preferentially at long intervals after the last feeding at the beginning of the dark cycle. After ~ 8 h fasting, Etruscan shrews start to enter torpor with a significant decrease in body temperature and activity. The surface body temperature of the Etruscan shrews was measured manually, taking thermal images using FLIR C5 thermal camera.

### Stereology

Coronal brain slices from male Etruscan shrews and rats were examined with StereoInvestigator software (MBF Bioscience, Wilistron, VT), employing an Olympus (Tokyo, Japan) BX5 1 microscope with an MBFCX9000 camera (MBF Bioscience) mounted on the microscope. The microscope was equipped with a motorized stage (LUDL Electronics, Hawthorne, CA) and a z-encoder (Heidenhain, Schaumburg, IL).

For estimating the hemispheric volume, we used Stereoinvestigator/Neurolucida software to draw the contour of the pia in every fourth slice of the Etruscan shrew and every twelfth slice of the rat under 10 × objective magnification. Contours of the MPN were drawn in each relevant slice for both Etruscan shrews and rats. We used Neurolucida/neuroexplorer software to calculate the cumulative surface area of all contours, and volumes were calculated by multiplying by the corresponding thickness of the slides.

For cell counting in the MPN, we employed a standard stereological sampling scheme called the optical fractionator method (MBL Stereoinvestigator). Our region of interest (MPN) was identified and outlined at low magnification, and neurons were identified by their shape and staining intensity at high objective magnification (100 × with oil). For counting, we evaluated the contours of the MPN in each slice containing the MPN in Etruscan shrews and for every second slice in rats. Optical dissectors were randomly placed on a series of sections, and we manually labelled the number of nucleoli that came into focus and lay within the defined lines of the dissector. This method provided an unbiased estimation because the number of neurons is estimated directly, without referring to neuron densities. We specifically used 15 × 15 µm counting frames and counted on average 0–4 neurons per frame.

### Statistics

Statistics were performed in Prism and Excel. For volumetric ratio analysis, brain samples from six male shrews and five rats were used and nonparametric Mann–Whitney *U* test was performed. We decided to reject the null hypothesis if the *p-*value is smaller than 0.01.

### Ethics statement

The study was conducted according to the guidelines of the ARRIVE. All experiments were performed according to German guidelines on animal welfare under the supervision of the local ethics committees and were approved by the Landesamt für Gesundheit und Soziales, Berlin; Permit numbers: G0170/15, T0160/14 andT0078/16.

## Supplementary Information

Supplementary Information.

## Data Availability

All data are provided within the manuscript or as Supplemental File.
